# Genomic Profiling of Antimicrobial Resistance Genes in Clinical *Salmonella* Isolates from Cattle in the Texas Panhandle, USA

**DOI:** 10.3390/antibiotics13090843

**Published:** 2024-09-04

**Authors:** Max Chung, Ethan Dudley, Hatem Kittana, Alexis C. Thompson, Matthew Scott, Keri Norman, Robert Valeris-Chacin

**Affiliations:** 1 College of Veterinary Medicine and Biomedical Sciences, Texas A&M University, Canyon, TX 79015, USA; chunmn26@exchange.tamu.edu (M.C.); edudley01@exchange.tamu.edu (E.D.); mascott@cvm.tamu.edu (M.S.); 2 College of Veterinary Medicine, Kansas State University, Manhattan, KS 66506, USA; hkittana@vet.k-state.edu; 3 Texas A&M Veterinary Medical Diagnostic Laboratory, Canyon, TX 79015, USA; alexis.thompson@tvmdl.tamu.edu; 4 College of Veterinary Medicine and Biomedical Sciences, Texas A&M University, College Station, TX 77843, USA; knorman@cvm.tamu.edu

**Keywords:** *Salmonella*, cattle, Texas, ARGs, bioinformatics, bacterial pathogenomics

## Abstract

Rising antimicrobial resistance (AMR) in *Salmonella* serotypes host-adapted to cattle is of increasing concern to the beef and dairy industry. The bulk of the existing literature focuses on AMR post-slaughter. In comparison, the understanding of AMR in *Salmonella* among pre-harvest cattle is still limited, particularly in Texas, which ranks top five in beef and dairy exports in the United States; inherently, the health of Texas cattle has nationwide implications for the health of the United States beef and dairy industry. In this study, long-read whole genome sequencing and bioinformatic methods were utilized to analyze antimicrobial resistance genes (ARGs) in 98 isolates from beef and dairy cattle in the Texas Panhandle. Fisher exact tests and elastic net models accounting for population structure were used to infer associations between genomic ARG profiles and antimicrobial phenotypic profiles and metadata. Gene mapping was also performed to assess the role of mobile genetic elements in harboring ARGs. Antimicrobial resistance genes were found to be statistically different between the type of cattle operation and *Salmonella* serotypes. Beef operations were statistically significantly associated with more ARGs compared to dairy operations. *Salmonella* Heidelberg, followed by *Salmonella* Dublin isolates, were associated with the most ARGs. Additionally, specific classes of ARGs were only present within mobile genetic elements.

## 1. Introduction

Salmonellosis is one of the leading causes of bacterial gastroenteritis in the United States, caused by non-typhoidal *Salmonella enterica* subspecies *enterica*. The Centers for Disease Control and Prevention (CDC) estimate that *Salmonella* accounts for 1.35 million cases and 26,500 hospitalizations in people annually [1]. Non-invasive infections are typically associated with mild cases of fever, diarrhea, vomiting, nausea, or abdominal cramps [2]. However, the symptoms can escalate if an infection becomes systemic, and complications such as meningitis, pancreatitis, or enteric fever that require antimicrobial intervention may ensue [3,4].

*Salmonella enterica* comprises over 2600 serotypes, but only a fraction of them are relevant in food production systems. Serotype-specific vaccination has been shown to reduce prevalence of dominant *Salmonella* serotypes in cattle, poultry, and swine, including Typhimurium, Enteritidis, Choleraesuis, and Dublin, among others [5]. However, vaccination may have promoted a disruption of serotype incidence, allowing other serotypes to replace the ecological niches [5].

The increased incidence of antimicrobial-resistant (AMR) bacteria is partially a product of antimicrobial mismanagement on a global scale. The issue is further exacerbated by the lack of new antibiotics to replace compounds with reduced efficacy. For example, one study estimated that resistance to clinically important antimicrobials increased 40% in *Salmonella* between 2004 and 2008 and 2015 and 2016 for human clinical infections in the United States [6].

Poultry and swine products are the primary sources of *Salmonella* in humans [7,8]. In the beef industry, economic resources and scientific efforts have traditionally been directed toward evaluating interventions in processing and packaging plants to reduce the risk of human salmonellosis from beef products [5,9]. However, the cattle industry is currently very interested in deepening our understanding of *Salmonella* ecology in pre-harvest cattle. In recent years, several regional studies have evaluated *Salmonella* in pre-harvest cattle or their immediate environment [5,10,11,12,13,14,15,16]. Additionally, recent reports have noted higher levels of resistance in *Salmonella* serotypes that are host-adapted to cattle such as *Salmonella* Dublin [17,18].

Some studies have evaluated AMR *Salmonella* and reported strong concordance between antimicrobial resistance genes and phenotypic resistance [10,19,20], prompting subsequent studies to assess AMR solely through genomic data [5,9,11,12,13,14,15,19,21]. Antimicrobial resistance gene prediction has been used to monitor antimicrobial resistance dynamics in *Salmonella* at the state level [5,11,19]. For instance, Carroll et al. (2020, 2021), carried out two experiments that relied solely on AMR genes to characterize *Salmonella* resistance for cattle in the state of New York [12,14]. These studies focused on groups of *Salmonella* serotypes to monitor herd population health, as antimicrobial resistance determinants are often serotype-associated [14]. Source tracking is also regularly applied in outbreak investigations to trace an outbreak back to a source so the corresponding regulations can be enforced to prevent future outbreaks [13,21]. More recently, the use of whole genome sequencing has been expanded to explore the role of mobile genetic elements on the dissemination of antimicrobial resistance genes to draw conclusions on resistance movement [5,15]. 

Knowledge gaps around antimicrobial resistance in *Salmonella* from cattle in Texas continue to exist despite the available literature [16,22,23,24,25]. No study has compared antimicrobial resistance in *Salmonella* from cattle between beef and dairy operations in Texas. Texas ranks the third largest in beef exports at USD 1.57 billion and fourth in dairy exports at USD 704 million [26]. The health of Texas cattle has nationwide implications for the resilience of beef and dairy food supply chains in the United States. To reduce the burden of salmonellosis in humans from food sources, a thorough understanding of the ecology of *Salmonella* in Texas cattle is necessary. Therefore, this study aimed to evaluate the distribution of antimicrobial resistance genes related to phenotypic resistance in *Salmonella* from beef and dairy cattle operations and potential associations with cattle age, sex, breed, specimen type, collection year, location, and *Salmonella* serotypes.

## 2. Results

### 2.1. Description of Salmonella Isolates

De novo genome assembly succeeded in 98 out of the 100 *Salmonella* isolates, which were used for downstream bioinformatics and statistical analyses. The isolates originated from cattle operations in Texas (n = 51), New Mexico (n = 15), or Arizona (n = 1) over a three-year period, i.e., 2021 (n = 35), 2022 (n = 38), and 2023 (n = 25), and were cultured from fecal (n = 77), intestinal (n = 19), or abomasal (n = 2) samples. Isolates originated from dairy operations (n = 71; n= 34 from dairies; n = 37 from calf ranches) and beef operations (n = 16; n = 11 from feedlots; n = 5 from cow-calf operations), and from dairy (n = 56) and beef breeds (n = 8). Isolates from male (n = 10) and female (n = 47) cattle, across different age groups, dichotomized as neonatal (n = 56) and not neonatal (n = 22), were included. Instances of missing data were present, as is usual with data from diagnostic laboratories. There were *Salmonella* isolates without information for sample origin (n = 31), cattle operation (n = 11), cattle breed (n = 34), sex (n = 41), and age (n = 20). Of the 31 *Salmonella* isolates without sample origin information, 25 isolates originated from submissions made by Texas-based practitioners, five by New Mexico-based practitioners, and one by a Louisiana-based practitioner. Due to the uncertainty of whether the location of the practitioner is a good proxy for the location of the sampled cattle, we did not use this additional information to replace the missing data for the sample origin. 

### 2.2. Nanopore Sequencing Bioinformatic Pipeline Performance

The long reads maintained an average quality score above Q20 for up to 20,000 bases. The GC content ranged between 51.88% to 52.36% of the genome. A total of 26 isolates assembled into one contig, 47 assembled in two or less, and 85 assembled in five or fewer. The mean NG50 and LG50 was 4,392,366.11 nucleotides and 1.05 contigs, respectively. 

Medaka polishing improved the quality of the assemblies as measured by the BUSCO scores. Prior to polishing the average complete, duplicated, fragmented, and missing BUSCO genes were 433.7, 1.46, 1.09, and 3.72, respectively. After polishing with Medaka the total number of complete BUSCO genes increased by 13 genes and missing or fragmented BUSCO genes decreased by 3 and 10 genes, respectively.

### 2.3. Overall Distribution of Phenotypic Antimicrobial Resistance among Salmonella Isolates

Guidelines from the Clinical & Laboratory Standards Institute (CLSI) supplement VET01S [27] for *Salmonella* restricted MIC interpretive standards to the following four antimicrobials (out of the 18 antimicrobials evaluated): ampicillin, gentamicin, tetracycline, and trimethoprim-sulfamethoxazole. Out of the 98 isolates, the highest levels of phenotypic resistance for the antimicrobials with CLSI breakpoints were observed in ampicillin and tetracycline, followed by trimethoprim-sulfamethoxazole and gentamicin (Table 1). Fourteen *Salmonella* isolates were resistant to three of the antimicrobials with CLSI breakpoints (ampicillin, tetracycline, trimethoprim-sulfamethoxazole), evidence of phenotypic multidrug resistance, since they are from different antimicrobial classes. Tetracycline resistance was observed among all isolates of *Salmonella* Bredeney, Cerro, Dublin, Heidelberg, and Meleagridis with the same being the case for ampicillin excluding *Salmonella* Meleagridis (Table 2). 

### 2.4. Antimicrobial Resistance Genes Detected in Salmonella Isolates

**Figure 1:**
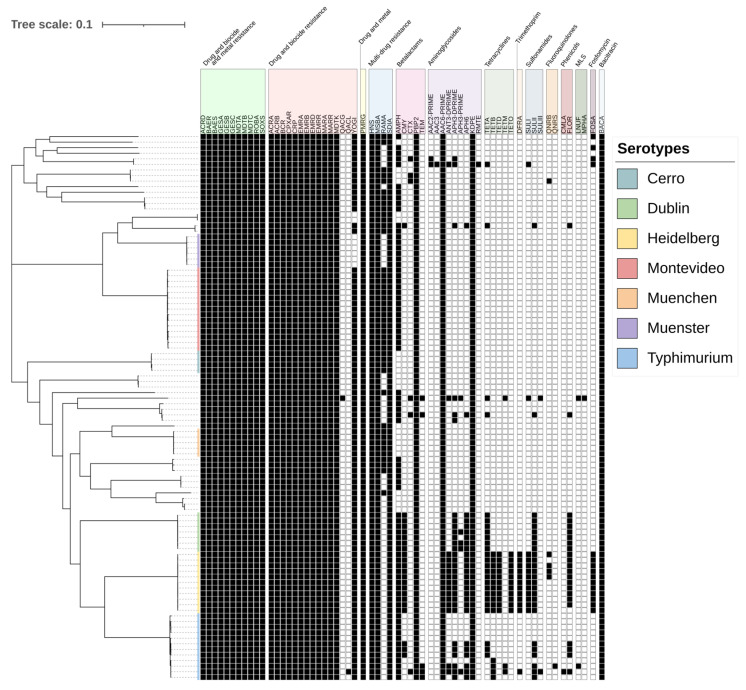
Maximum likelihood phylogeny of 98 *Salmonella* isolated from cattle in the Texas Panhandle. The colors in the phylogeny represent the six most common serotypes from SISTR [28]. The table summarizes the presence (black) or absence (white) of resistance for each isolate (row). Antimicrobial resistance genes were grouped by resistance class. MLS: macrolides, lincosamides, and streptogramines. The scale bar represents the average nucleotide substitution per site in the phylogeny tree.

The *Salmonella* isolates harbored 14 classes of antimicrobial resistance genes (Figure 1). Resistance genes followed serotype patterns. *Salmonella* Heidelberg carried the largest set of antimicrobial resistance classes. Trimethoprim resistance genes were only found in *Salmonella* Heidelberg, apart from one *Salmonella* Typhimurium isolate. Fluoroquinolone, fosfomycin, and tetracycline resistance genes were also most prevalent among *Salmonella* Heidelberg isolates. 

*Salmonella* Dublin and *Salmonella* Heidelberg genomes shared many antimicrobial resistance genes. Sulfonamide and phenicol resistance genes were identified in all genomes of those serotypes. Both serotypes along with Typhimurium also carried an array of aminoglycoside resistance genes.

### 2.5. Mapping Antimicrobial Resistance Genes to Plasmids

Thirty genomes carried plasmids typically associated with antimicrobial resistance genes. Several antimicrobial resistance genes were mapped to the same contigs as the plasmids (Table 3). Of notice, none of the isolates harbored mobile colistin-resistance genes (*mcr*). Among the 11 *Salmonella* Heidelberg genomes, all featured an IncA/C2 plasmid, six carried resistance pattern two, two carried resistance pattern four, and the remaining three had similar sets of genes with a few modifications (patterns one, three, and five) (Table 3). Additionally, a col440I plasmid with a *qnrB* gene was found in addition to the IncA/C2 plasmid in four *Salmonella* Heidelberg genomes, including two genomes with resistance pattern two, one with resistance pattern three, and one with resistance pattern four. One of three *Salmonella* Cannstatt genomes was also found to contain a col440I plasmid with the *qnrB* gene.

Two incompatibility groups characterized *Salmonella* Typhimurium isolates. Three genomes contained an IncA/C2 plasmid with resistance pattern seven, while the remaining two isolates carried resistance genes on an IncFIB plasmid, one with resistance pattern six and the other with resistance pattern eight. The former also contained a colRNAI plasmid with the resistance genes *aph(3″)*, *aph(3′)*, and *bla*_CTX-M_.

The IncA/C2 plasmid was responsible for all three resistance patterns detected in *Salmonella* Dublin isolates. Three isolates had resistance pattern 10, two had resistance pattern 9 and two had resistance pattern 7. One of two *Salmonella* Bredeney genomes also carried an IncA/C2 plasmid with resistance pattern 12.

Both *Salmonella* Meleagridis genomes carried resistance plasmids. One carried a colRNAI plasmid with the resistance genes *aph(3″)*, *aph(3′)*, and *bla*_CTX-M_, while the other had an IncHI2 plasmid with resistance pattern 11. A *Salmonella* Uganda isolate was also found to contain an IncHI2 plasmid with resistance pattern 13. Two *Salmonella* Anatum isolates carried resistance plasmids. One genome included an IncR plasmid with resistance pattern 12, and the other carried a colRNAI plasmid with *aph(3″)*, and *aph(3′)*. 

### 2.6. Antimicrobial Resistance Genes Significantly Associated with Serotypes

**Figure 2:**
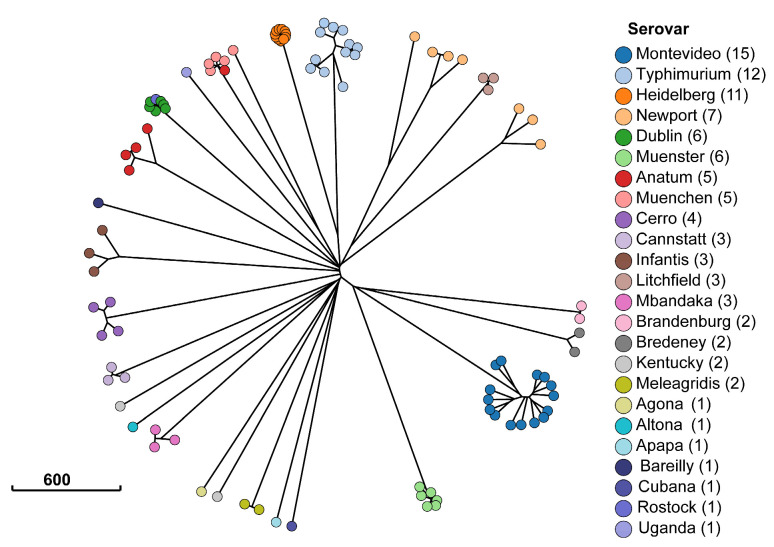
Minimum spanning tree of Enterobase sequence typing [33]. Each dot represents one of the 98 *Salmonella* isolates from cattle in the Texas Panhandle. The colors indicate the serotype classification from SISTR [28] and the scale bar indicates the number of allele sequence type differences between isolates in the *Salmonella* cgMLST scheme.

This study identified a total of 23 serotypes in silico. The results for core genome multilocus sequencing typing (cgMLST) are illustrated in Figure 2. *Salmonella* isolates clustered mostly by serotype, with the exception of one *Salmonella* Anatum isolate clustering with the *Salmonella* Muenchen isolates, and one *Salmonella* Rostock isolate clustering with the *Salmonella* Dublin isolates. However, only four *Salmonella* serotypes were statistically significantly associated with antimicrobial resistance genes. Table 4 highlights the genes statistically significantly associated with serotypes. *Salmonella* Heidelberg carried the largest set of antimicrobial resistance genes, with 12 out of 14 genes exhibiting complete separability (the gene predicted the phenotype perfectly). Among the statistically significant genes, 70% belonged to genes encoding for resistance against aminoglycosides, sulfonamides, or tetracyclines. *Salmonella* Dublin was the second most significant reservoir for antimicrobial resistance genes. In addition to sharing six genes with *Salmonella* Heidelberg, the odds of carrying *aph(3′)* was 33.4-times higher among *Salmonella* Dublin isolates compared to all other serotypes (*p* = 0.01).

### 2.7. Antimicrobial Resistance Genes Associated with Salmonella Isolates from Beef and Dairy Operations

**Figure 3:**
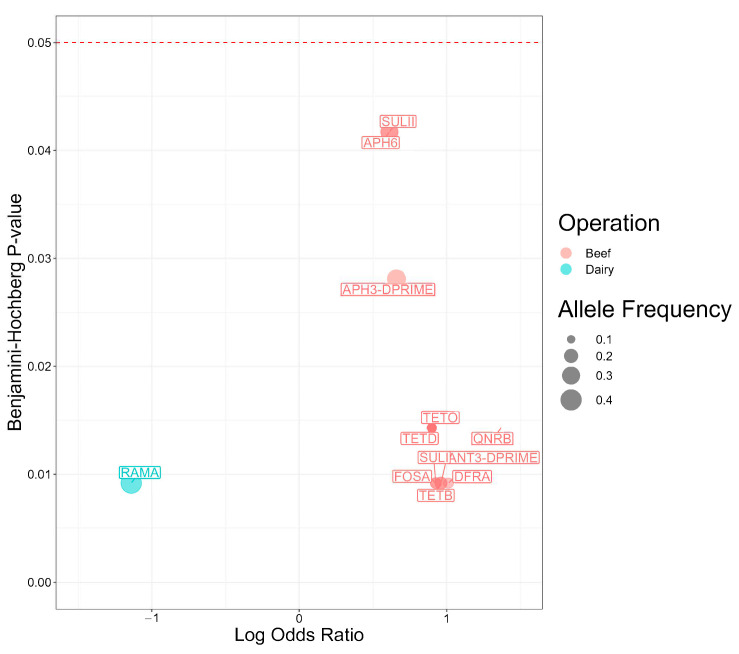
Antimicrobial resistance genes present in 98 *Salmonella* isolates from Texas Panhandle cattle associated with beef or dairy operations (Scoary analysis). Log Odds Ratios: natural log-transformed odds ratios were obtained from a main effects model and adjusted for population structure. Benjamini–Hochberg *p*-value: *p* value adjusted for false discovery rate using the Benjamini-Hochberg method.

The findings for the Scoary analysis for genes significantly associated with beef or dairy operations are shown in Figure 3. Among the 12 statistically significant genes, 11 exhibited stronger associations with *Salmonella* isolates from beef operations. The *qnrB* gene had the strongest association (*p* = 0.014). The odds of carrying the gene were 23.3-times higher in *Salmonella* isolates from beef operations than dairy.

Genes encoding for resistance against aminoglycosides emerged as the most prevalent class of antimicrobial resistance genes found in *Salmonella* isolates from beef operations. The odds for carrying aminoglycoside resistance genes were 9.1-times higher for *ant*(3″), 4.6-times higher for *aph(3″)*, and 4.1-times higher with *aph(6)* in *Salmonella* isolates from beef operations compared to dairy (*p* = 0.009, 0.028, and 0.042, respectively).

Two sulfonamide resistance genes exhibited statistical associations with beef operations. The odds ratio of carrying *sul1* (OR = 8.4, *p* = 0.009) was twice the odds ratio of carrying *sul2* (4.1, *p* = 0.042) in beef operations compared with dairy operations. Given trimethoprim is typically administered in conjunction with sulfonamides to treat salmonellosis, the odds of harboring *dfrA* were 10.3-times higher in *Salmonella* isolates from cattle on beef operations compared to dairy operations (*p* = 0.009). 

Multiple tetracycline resistance genes were statistically significantly associated with beef operations. The odds of carrying *tet(*B*)* were 9.1-times higher in *Salmonella* isolates from cattle on beef operations (*p* = 0.009) and the odds of carrying *tet(*D*)* or *tet(*O*)* were 7.9-times higher (*p* = 0.014). The gene *ramA* was the only gene with a statistically significant association with dairy operations. The odds of carrying *ramA* were 92.7% higher in *Salmonella* isolates from cattle on dairy operation than beef operations (*p* = 0.009).

**Figure 4:**
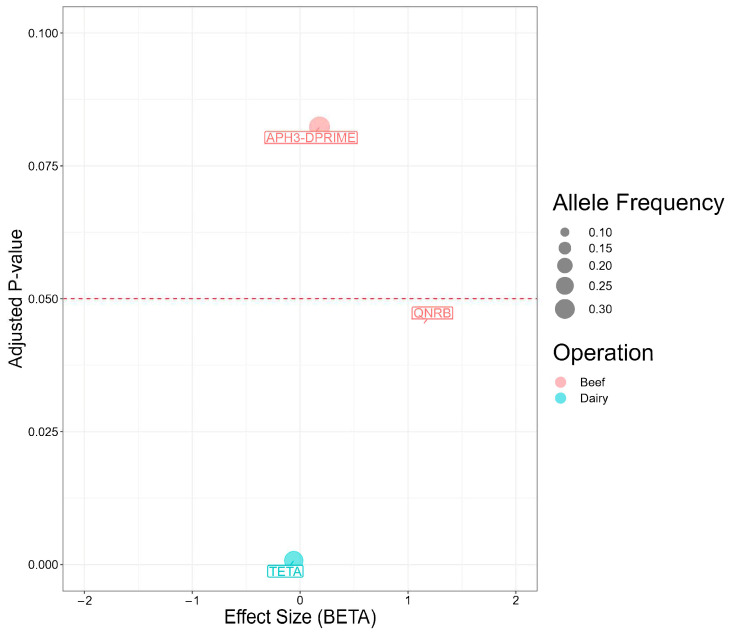
Antimicrobial resistance genes present in 98 *Salmonella* isolates from Texas Panhandle cattle associated with beef or dairy operations (Pyseer analysis). Effect size (BETA) represents the natural log-transformed odds ratio (that is the coefficient of the penalized regression) adjusted for overfitting, population structure, and the other antimicrobial resistance genes in the model. Adjusted *p*-value: *p* value adjusted for population structure.

Given the nature of penalized logistic regression, Pyseer analysis yielded a truncated set of genes associated with cattle operations (Figure 4). Among these, two genes were statistically significant. Notably, the odds of originating in a beef operation were 3.2-times higher in *Salmonella* isolates carrying *qnrB* compared to isolates without the gene (*p* = 0.045). By contrast, the odds of originating in a dairy operation were 6% higher in *Salmonella* isolates carrying *tet(*A*)* (*p* = 0.0007).

### 2.8. Association between Antimicrobial Resistance Genes and Antimicrobial Susceptibility Testing

**Figure 5:**
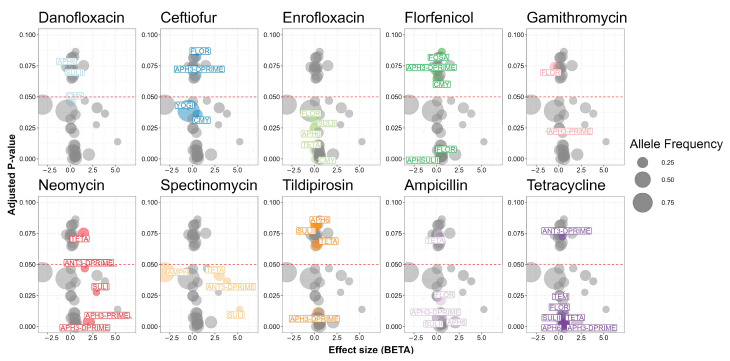
Antimicrobial resistance genes in 98 *Salmonella* isolates from Texas Panhandle cattle associated with phenotypic antimicrobial resistance. Effect size (BETA) represents the natural log-transformed odds ratio (that is the coefficient of the penalized regression) adjusted for overfitting, population structure, and the other antimicrobial resistance genes in the model. Adjusted *p*-value: *p* value adjusted for population structure.

Pyseer detected associations between antimicrobial resistance genes and antimicrobial susceptibility results (Figure 5). The phenotypic resistance to 10 out of 18 antimicrobials had significant associations with resistance genes. For five antimicrobials a significant association between the phenotypic resistance and the corresponding antimicrobial resistance gene was observed. 

Phenotypic resistance to neomycin had the most biologically relevant significant associations with antimicrobial resistance genes. Two aminoglycoside resistance mechanisms were identified. One aminoglycoside O-nucleotidyltransferase was statistically significant (*p* = 0.0469). The MIC of neomycin was 1.6 units higher in *Salmonella* isolates carrying *ant(3″)* than isolates not carrying the gene. Two aminoglycoside O-phosphotransferase genes also had associations with neomycin. The MIC of neomycin was 2.1 or 6.8 units higher for *Salmonella* isolates carrying *aph(3″)* or *aph(3′)* than isolates not carrying either gene (*p* = 0.0036 and 0.0066, respectively).

Ceftiofur was the only antimicrobial with a statistically significant positive and negative association. The MIC for ceftiofur was 0.6 units higher in isolates carrying *bla*_CMY_, but 0.4 units lower in isolates carrying *yogi* compared to isolates not carrying either gene (*p* = 0.0357 and 0.0388, respectively).

Single antimicrobial resistance genes drove resistance against three antimicrobials. The MIC for florfenicol was 0.6 units higher in *Salmonella* isolates carrying *floR* than isolates not carrying the gene (*p* = 0.00327). The MIC for spectinomycin was 3.8 units higher in *Salmonella* isolates carrying *ant(3″)* than isolates not carrying the gene (*p* = 0.0365). The odds of being resistant to tetracycline were 95% higher among *Salmonella* isolates carrying *tet*(A) compared to isolates not carrying *tet*(A) (*p* = 0.002910). 

Scoary detected biologically relevant associations with phenotypic resistance in three of the four antimicrobials with MIC interpretation. Tetracycline had the largest number of genes. The *tet*(A), *tet*(B), *tet*(D), *and tet*(O) genes all showed complete separability for isolates classified as resistant. The *bla*_CMY_ gene was also found to have complete separability with the ampicillin resistance phenotype. 

Three genes were statistically significantly associated with resistance to trimethoprim-sulfamethoxazole. One gene confers resistance to trimethoprim. The odds of carrying *dfrA* was 33.9-times higher among *Salmonella* isolates resistant to trimethoprim-sulfamethoxazole (*p* < 0.0001). Two genes that confer resistance to sulfamethoxazole were also statistically significant. The odds of carrying *sul1* or *sul2* were 25.1- and 9.8-times higher among *Salmonella* isolates resistant to trimethoprim-sulfamethoxazole (*p* < 0.0001 and 0.0004, respectively).

### 2.9. Other Associations

Pyseer also found associations between antimicrobial resistance genes and the remaining phenotypes. The gene *tet*(B) was the only one with geographical associations. The odds of originating from New Mexico (versus Texas or Arizona) were 1.9-times higher in *Salmonella* isolates carrying *tet*(B) compared to those without the gene (*p* = 0.0499); additionally, the odds of originating in Texas (versus New Mexico or Arizona) were 4.5-times lower in *Salmonella* isolates carrying *tet*(B) compared to the isolates without the gene (*p* = 0.0466). Cattle sex was also linked to antimicrobial resistance genes. The odds of a cattle being male were 28.2-times higher in *Salmonella* isolates carrying *fosA* compared to isolates without the gene (*p* = 0.0482), while the odds of cattle being female were 5.3-times higher in *Salmonella* isolates carrying *ampH* compared to isolates not carrying the gene (*p* = 0.00912). Three genes had negative associations with *Salmonella* isolates collected in 2022. The odds of isolates being collected in 2022 (versus in 2021 or 2023) were 74%, 12%, and 75% lower in *Salmonella* isolates carrying *aph(6)*, *floR*, and *sul2*, respectively, than in isolates without those genes (*p* = 0.0081, 0.0307, and 0.0081, respectively). 

## 3. Discussion

This study aimed to identify antimicrobial resistance genes in *Salmonella* from cattle residing in the Texas Panhandle region. The results of this study highlighted the differences in the prevalence of antimicrobial resistance genes in *Salmonella* between beef and dairy operations and serotypes. *Salmonella* isolated from cattle in beef operations had more antimicrobial resistance genes. *Salmonella* Heidelberg, followed by *Salmonella* Dublin, harbored the most antimicrobial resistant genes. Additionally, specific classes of antimicrobial resistance genes were only present within mobile genetic elements.

Several studies have reported strong concordance between antimicrobial resistance genes and resistance phenotypes in the past [10,19,20,34]. For example, Carroll et al. (2017) reported a prediction sensitivity of 97.2% and a specificity of 85.2% [10]. However, the statistical methods employed by these studies do not adjust for common confounders in bacterial genome-wide association studies such as population structure [35,36]. In our study, only five antimicrobials resistance determinants (against ceftiofur, florfenicol, neomycin, spectinomycin, and tetracycline) were significantly associated with higher MICs for the corresponding antimicrobials. We attribute these discrepancies, in part, to the differences in the bioinformatics processing and statistical analysis. The presence or absence of a resistance gene is not the sole indicator of resistance. Enzyme activation, target modification, gene expression regulation, or cell wall configuration changes also influence phenotypic resistance [20,37]. Antimicrobial resistance is a multifactorial problem involving management practices and mineral deficiency, in addition to transmission of antimicrobial resistant bacteria [38,39,40,41,42].

*Salmonella* isolates carrying IncA/C2 plasmids often originate from cattle sources [9,43]. The rise of multidrug-resistant *Salmonella* in 2010 coincided with the emergence of IncA/C2 plasmids [44,45]. In this study, IncA/C2 plasmids were responsible for conferring resistance in most (22 of 30) isolates for all antimicrobial resistance genes that predicted the correct corresponding antimicrobial resistance phenotype. This is not surprising as antimicrobial resistance stemming from IncA/C2 has been often reported in *Salmonella* [19,46,47,48]. 

A quinolone gene was only present on col440I plasmids. Plasmid-mediated quinolone resistance (PMQR) is well characterized in *Salmonella*. Col plasmids often carry PMQR genes that mediate reduced susceptibility to quinolones in Enterobacteriaceae, but they do not typically manifest into resistance [49,50,51,52]. While identifying point mutations was beyond the scope of this study, resistance is not achieved without point mutations in the quinolone resistance determining region of *parC* or *gyrA* [9,10,16,19,53,54,55]. Additional point mutations lead to higher levels of phenotypic resistance [45,56,57].

*Salmonella* serotypes often follow particular antimicrobial resistance patterns, and many factors can be associated with their prevalence and distribution. A study by Levent et al. (2019) found that the pen was the most important factor contributing to the prevalence of specific serotypes in cattle herds [22]. Given our study acquired isolates through convenience sampling, they did not originate from the same operation and therefore we cannot evaluate the pen effect. However, we similarly observed that antimicrobial resistance patterns were associated with specific serotypes. *Salmonella* Dublin and *Salmonella* Heidelberg harbored the most antimicrobial resistance genes. Both serotypes are of high concern because they are responsible for the third (0.31) and fourth (0.27) highest hospitalization to illness ratios among non-typhoidal *Salmonella* infections [58]. The rise of multidrug-resistant *Salmonella* Dublin, the most prominent serotype found in clinical cases from cattle, has been documented to have arisen from the emergence of IncA/C2 plasmids [17,37,45,59,60]. Our study only detected *Salmonella* Dublin from dairy operations and *Salmonella* Dublin has been reported to have increasing prevalence in dairy facilities [17]. Interestingly, we did not find genotypic multidrug resistance in either *Salmonella* Cerro or *Salmonella* Montevideo, which represent over 27% of *Salmonella* isolates in bovine cases [45]. Pan-susceptibility from *Salmonella* Cerro and *Salmonella* Montevideo is consistent with the existing literature on cattle [61,62,63].

In recent decades, *Salmonella* Heidelberg has broadened its resistance profile, leading to increased hospitalizations in people [13]. The relationship between elevated virulence and co-selection with antimicrobial resistance may be responsible for the virulence factors and antimicrobial resistance genes consistently expressed by this serotype [13,45,64,65]. In our study, *Salmonella* Heidelberg was identified in cattle on both beef and dairy cattle operations. The multidrug resistant phenotype in this serotype is commonly associated with dairy beef calves [66].

*Salmonella* Infantis is well known for exhibiting multidrug resistance in poultry [67]. In our study, all three isolates were phenotypically susceptible to the four antimicrobials with CLSI breakpoints but harbored an important set of antimicrobial resistance genes, suggesting they may be multidrug-resistant isolates, findings consistent with the existing literature [68,69]. 

Host age is a major contributing factor to the prevalence of antimicrobial resistance in *Salmonella*. Bacteria recovered from dairy calves often exhibit higher levels of antimicrobial resistance than bacteria from adults in part due to early exposure to antimicrobials as prevention measures against diseases [38,39,40,70,71,72,73]. Administration of antimicrobials is limited during lactation to prevent antimicrobial residues from contaminating milk, leading to lower level of antimicrobial resistance genes in *Salmonella* from adult dairy cows [74,75]. However, cows in dry off are often administered antimicrobials to manage mastitis and protect the performance of future lactation cycles [76,77]. 

Hille et al. (2017) found that less rigorous practices led to lower prevalence of antimicrobial resistant bacteria in beef operations, which Tello et al. (2020) claims is due to lower stress and infection pressure [74,78]. Similar to dairy practices, antimicrobials are also used sparingly in beef cattle approaching slaughter because if antimicrobials are administered close to slaughter, additional resources are required to support an extended withdrawal period to prevent antimicrobial residues in the meat [79]. We did not find any significant associations between antimicrobial resistance genes and the age of cattle. We attribute this lack of association to sparsity of the metadata. Cattle could only be dichotomized based on neonatal status (age < 1 month) and even then 20% of samples were excluded due to missing data. 

Recent studies have reported differentially abundant antimicrobial resistance genes in the resistomes of beef compared to dairy cattle. Rovira et al. (2019) reported that beef cattle feces had more relatively abundant antimicrobial resistance genes than dairy cattle feces, but relative abundance differed by resistance class. Beef cattle resistomes featured a higher relative abundance of genes conferring resistance to tetracycline and macrolides, but dairy cattle resistomes had higher relative abundance of beta-lactamases [80]. Wang et al. (2021) also found that beef cattle gut samples carried more antimicrobial resistance genes than dairy. Beef cattle resistomes had a higher relative abundance of macrolides, beta-lactamases, and multidrug resistance genes and dairy cattle resistomes had more quinolone and aminoglycoside resistance genes [81]. While the resistome of cattle feces was not the focus of this study, we also observed that *Salmonella* isolates from beef operations showed disproportionally more associations with antimicrobial resistance genes than those from dairy operations with both statistical approaches. However, associations with *Salmonella* from beef and dairy operations could have been confounded by age, considering most isolates from beef operations did not have a reported age (9/16), and three out of the seven isolates with age information were from calves under one month old. 

This study contributes to federal food safety initiatives to reduce *Salmonella* in the beef industry continuum, particularly in the pre-harvest stage, which is underrepresented relative to poultry or swine in research. However, this study does have limitations. The isolates from this study originated from sample submissions positive for *Salmonella*. Consequently, there may be selection bias because no isolates from healthy hosts were included. Since it was a convenience sampling, it is difficult to generalize findings to the cattle populations of the Texas Panhandle cattle. Additionally, the sample size was small, and the diversity of the isolates was constrained to what clients submitted to the Texas A&M Veterinary Medical Diagnostic Laboratory, leading to a disproportionate number of isolates from dairy operations. Many samples also were not submitted with complete descriptions of the sample’s origin because the level of detail for the metadata was at the will of the client, creating instances of missing data. We could not determine whether the submissions were related to clinical salmonellosis cases. The search for antimicrobial resistance genes was conducted with a minimum percent identity and coverage of 80%, which may have influenced the detection of some antimicrobial resistance genes not commonly reported in *Salmonella*. However, 42% of genes had a coverage of 100% and an identity of 90%. Future studies should adopt probabilistic sampling approaches that include a representative sampling of all stages of pre-harvested beef and dairy production to gain a deeper understanding of the ecology of *Salmonella* from cattle in Texas.

## 4. Materials and Methods

### 4.1. Isolate Selection and Antimicrobial Susceptibility Testing

A total of 100 *Salmonella* isolates that were recovered from different bovine enteric samples (intestinal tissues, feces or fecal swabs) were used in this study. All specimens were obtained from clinically ill cattle and submitted to the American Association of Veterinary Laboratory Diagnosticians (AAVLD)-accredited Texas A&M Veterinary Medical Diagnostic Laboratory at Canyon, TX, USA (TVMDL-Canyon) for culture. Submissions ranged between the years 2021 (n = 36), 2022 (n = 38), and 2023 (n = 26). Isolation procedures, biochemical characterization, and detection of different *Salmonella* isolates were performed utilizing well-established morphological and biochemical identification methods for *Salmonella* at the TVMDL-Canyon [82,83,84]. Antimicrobial susceptibility testing for all 100 isolates was performed using broth microdilution MIC plate method (Sensititre™ Vet Bovine BOPO7F Plate; ThermoFisher, Waltham, MS, USA), following the manufacturer’s instructions [85,86] at the TVMDL-Canyon. Antimicrobial resistance patterns (phenotypes) for *Salmonella* isolates were determined by the BIOMIC V3 (Giles Scientific, Santa Barbara, CA, USA) instrument employing the most recently breakpoints published by CLSI in the VET01S supplement for accurate analysis [27,87,88]. Frozen *Salmonella* isolates were transferred to the Texas A&M Veterinary Education, Research, & Outreach (VERO) Research Laboratory (Canyon, TX, USA ) for bacterial DNA extraction and whole genome sequencing.

### 4.2. Bacteriological Culture

*Salmonella* isolates underwent a series of microbiological culture steps to confirm the identity of *Salmonella* and assess the purity of the isolates. Isolates were inoculated in Brain Heart Infusion (BHI) broth (Becton Dickinson, Franklin Lakes, NJ, USA) and incubated for 24 h at 37 °C under aerobic conditions. If bacterial growth was evidenced via turbidity, an aliquot was subcultured onto Xylose-Lysine-Tergitol 4 (XLT4) (Hardy Diagnostics, Santa Maria, CA, USA) and MacConkey (Hardy Diagnostics) agar plates, and incubated for 24 h at 37 °C under aerobic conditions to confirm the presence of *Salmonella*. Isolates with *Salmonella*-like colonies (black colonies on XLT4 and pale colonies on MacConkey agar plates) were subcultured to assess their purity, picking one colony from the XLT4 plate and streaking it onto a Columbia blood agar plate (Hardy Diagnostics) with 24-hour incubation at 37 °C under aerobic conditions. One colony per isolate was inoculated from Columbia blood agar plate into BHI broth and incubated for 24 h at 37 °C under aerobiosis before DNA extraction.

### 4.3. DNA Extraction

Each pure *Salmonella* isolate in BHI broth was centrifuged in 1.5 mL vials at 10,000× *g* for 1 min at 4 °C in two consecutive rounds (using 1 mL and 0.5 mL of BHI broth, respectively) to pellet the bacterial cells before extracting the DNA. DNA was extracted using the DNeasy Ultraclean Microbial Kit on a QIAcube Connect RNA/DNA extraction instrument (Qiagen, Hilden, Germany) using the manufacturer’s instructions. The heat treatment (65 °C for 10 min) was chosen instead of the bead beating step as recommended by the manufacturer to reduce DNA shearing. 

### 4.4. Whole Genome Sequencing

The extracted DNA (800–1000 ng per isolate) was sheared using g-TUBES (Covaris, Woborn, MA, USA) to obtain an expected fragment length of 8 kbp, following manufacturer’s instructions. The sheared DNA (in 150 μL of low EDTA TE buffer) was cleaned using AMPure XP beads (Beckman Coulter, Brea, CA, USA) at a ratio of 0.4× and eluted in 14 μL of molecular grade water (Corning, Corning, NY, USA). The sheared DNA was assessed via visualization of the electropherogram in a genomic ScreenTape (Agilent, Santa Clara, CA, USA) and its concentration and quality were evaluated using a NanoDrop Eight (ThermoFisher, Waltham, MA, USA). DNA libraries were prepared using ONT Native Barcoding Kit V14 (SQK-NBD114-24; ONT, Oxford, UK), multiplexing 24 *Salmonella* isolates per DNA library. Three extraction blanks were also processed for sequencing to assess the presence of background DNA. Sequencing was performed on a MinION Mk1C (ONT) device with a flow cell version 10.4.1 at a speed of 260 bases per second for 72 h, using the Fast model (MinKNOW version 22.10.7, Guppy version 6.3.9) due to the constraints in the computational resources of the device. After the sequencing run, the corresponding FAST5 files were base called again with the Super Accurate Model (standalone Guppy version 6.5.7) on a dedicated workstation for downstream analysis. 

### 4.5. Bioinformatics and Statistical Analysis

The read quality was assessed with LongQC (version 1.2.0) [89], and long reads were assembled using Flye (version 2.9.1) [90]. In those cases where Flye failed to assemble the *Salmonella* genome, Canu (version 2.2) [91] was used. Genomes were first polished with Medaka (version 1.7.2) [92], followed by a second round of polishing with Homopolish (version 0.4.1) [93]. Afterward, QUAST (version 5.0.2) [94] and BUSCO (version 5.1.2) [95] were employed to evaluate the structure and biological relevancy of the assemblies using serotype-specific references from NCBI, matching the serotype classification of the isolates obtained in silico with the *Salmonella* In Silico Typing Resource (SISTR, version 1.1.2) [28]. *Salmonella* isolates having assemblies with coverages below 60× were re-extracted and re-sequenced to obtain higher coverage. 

The phylogenetic relationship between isolates was assessed with two methods. Samples were sequenced typed using the Enterobase *Salmonella* cgMLST scheme (version 1.2.0) [33] according to allele variant matching with 100% identity to the housekeeping sequences. Additionally, a SNP-based maximum likelihood phylogeny was generated using Snippy (version 4.6.0) [96] and Molecular Evolutionary Genetics Analysis (MEGA) 11 with the Tamura-Nei substitution model and the Nearest-Neighbor-Interchange maximum likelihood heuristic method [97]. ABRicate (release FOFN MOTD 80 80) [30] was employed to search for antimicrobial resistance genes with the MEGARes database (version 2.00) [31]. The genes detected by ABRicate were evaluated against the available metadata (sample type, cattle operation, cattle breed, age, sex, sample origin, and collection year) and antimicrobial susceptibility profiles (ampicillin, ceftiofur, clindamycin, danofloxacin, enrofloxacin, florfenicol, gamithromycin, gentamicin, neomycin, penicillin G, spectinomycin, sulfadimethoxime, tetracycline, tildipirosin, tilmicosin, trimethoprim-sulfamethoxazole, tulathromycin, and tylosin).

The associations between the antimicrobial resistance genes and the metadata were evaluated with two statistical approaches. Scoary (version 1.6.16) [98] was utilized to detect associations between individual genes with each variable in the metadata using a Fisher’s exact test, while controlling the false discovery rate with the Benjamini–Hochberg method according to a *p*-value cutoff of 0.05 and adjusting for the population structure from the maximum likelihood phylogeny described above. An elastic net logistic regression in Pyseer (version 1.3.11) [99] was also applied to assess the independent associations between the genetic determinants and each outcome, adjusting for the population structure using the maximum likelihood phylogeny described above. Isolates with intermediate resistance were reassigned as susceptible for the analysis. The MICs of the antimicrobials without CLSI breakpoints were assessed with elastic net linear regression, also in Pyseer.

Bandage was used to annotate and map antimicrobial resistance genes and plasmid sequences (from ABRicate with the MEGARes and PlasmidFinder [32] databases, respectively) to Flye and Canu assemblies. In Bandage, BLAST hits were restricted to hits with a query identity and coverage above 80%. In cases where more than one plasmid mapped to a contig, the plasmid sequence with the highest identity and coverage was selected as the consensus [29].

## 5. Conclusions

This study summarizes antimicrobial resistance genes found in 98 *Salmonella* isolates from cattle residing in the Texas Panhandle. Antimicrobial resistance genes did not explain all antimicrobial resistance phenotypes. Some antimicrobial resistance genes were present in mobile genetic elements. A few serotypes displayed multidrug resistance and antimicrobial resistance gene profiles differed in beef operations compared to dairy operations. Based on this study, mitigation strategies for multidrug resistant *Salmonella* should continue to be developed for the beef industry to help curb the rising antimicrobial resistance trends.

## Figures and Tables

**Table 1:** Antimicrobial susceptibility in *Salmonella* isolates from cattle in the Texas Panhandle for antimicrobials with CLSI clinical cutoffs (n = 98).

Antimicrobial	Susceptible	Intermediate	Resistant
Ampicillin	39 (40)	1 (1)	58 (59)
Gentamicin	94 (96)	1 (1)	3 (3)
Tetracycline	38 (39)	3 (3)	57 (58)
Trimethoprim-sulfamethoxazole	82 (84)	0 (0)	16 (16)

Values within parentheses are percentages. Cutoffs from Clinical & Laboratory Standards Institute supplement VET01S [27].

**Table 2:** Antimicrobial resistance among *Salmonella* serotypes from cattle in the Texas Panhandle (n = 98 isolates) *.

Serotype	N	Tetracycline	Ampicillin	Gentamicin	Trimethoprim-Sulfamethoxazole
Montevideo	15	7 (47)	7 (47)	1 (7)	2 (13)
Typhimurium	12	8 (67)	9 (75)	0	10 (83)
Heidelberg	11	11 (100)	11 (100)	0	9 (82)
Dublin	7	7 (100)	7 (100)	0	1 (14)
Newport	7	2 (29)	2 (29)	0	0
Muenchen	6	4 (67)	4 (67)	0	1 (17)
Muenster	6	3 (50)	2 (33)	0	1 (17)
Cerro	4	4 (100)	4 (100)	0	0
Anatum	4	1 (25)	1 (25)	0	0
Litchfield	3	1 (33)	1 (33)	0	0
Cannstatt	3	1 (33)	1(33)	1 (33)	0
Infantis	3	0	0	0	0
Mbandaka	3	1 (33)	1 (33)	0	1 (33)
Bredeney	2	2 (100)	2 (100)	0	0
Brandenburg	2	0	0	0	0
Kentucky	2	1 (50)	1 (50)	0	0
Meleagridis	2	2 (100)	1 (50)	1 (50)	0
Uganda	1	1 (100)	1 (100)	0	0
Cubana	1	1 (100)	1 (100)	0	0
Apapa	1	0	1 (100)	0	0
Altona	1	0	1 (100)	0	0
Algona	1	0	0	0	0
Bareilly	1	0	0	0	0

* For antimicrobials with clinical cutoffs from the Clinical & Laboratory Standards Institute (CLSI) supplement VET01S [27]; values within parentheses are percentages.

**Table 3:** Antimicrobial resistance genes constituting resistance patterns for mobile genetic elements in 98 *Salmonella* isolates from cattle in the Texas Panhandle.

Resistance Pattern	Serotype	Plasmid Replicon	Antimicrobial Resistance Genes
1	Heidelberg	IncA/C2	*ant(3″)*, *aph(3″)*, *aph(3′)*, *aph(6)*, *bla*_BIL_, *bla*_CFE_, *bla*_CMY_, *bla*_LAT_, *dfrA*, *qacE*, *qacEdelta1*, *sul1*, *sul2*, *tet(*A*)*, *tet(*B*)*, *tet(*D*)*, *tet(*O*)*
2	Heidelberg	IncA/C2	*ant(3″)*, *aph(3″)*, *aph(3′)*, *aph(6)*, *bla*_BIL_, *bla*_CFE_, *bla*_CMY_, *bla*_LAT_, *dfrA*, *floR*, *qacE*, *qacEdelta1*, *sul1*, *sul2*, *tet(*A*)*, *tet(*B*)*, *tet(*D*)*, *tet(*O*)*
3	Heidelberg	IncA/C2	*aac(6′)*, *ant(2″)*, *ant(3″)*, *aph(3″)*, *aph(3′)*, *aph(6)*, *bla*_BIL_, *bla*_CARB_, *bla*_CFE_, *bla*_CMH_, *bla*_CMY_, *bla*_CTX-M_, *bla*_IMP_, *bla*_LAT_, *bla*_MOX_, *bla*_OXA_, *bla*_VEB_, *bla*_VIM_, *dfrA*, *floR*, *ges*, *qacE*, *qacEdelta1*, *sul1*, *sul2*, *tet32*, *tet(*A*)*, *tet(*B*)*, *tet(*D*)*, *tet(*O*)*, *tet(*W*)*
4	Heidelberg	IncA/C2	*ant(3″)*, *aph(3″)*, *aph(6)*, *bla*_BIL_, *bla*_CFE_, *bla*_CMY_, *bla*_LAT_, *dfrA*, *floR*, *qacE*, *qacEdelta1*, *sul1*, *sul2*, *tet(A)*, *tet(*B*)*, *tet(*D*)*, *tet(*O*)*
5	Heidelberg	IncA/C2	*ant(3″)*, *aph(3″)*, *aph(6)*, *bla*_CFE_, *bla*_CMY_, *bla*_LAT_, *dfrA*, *floR*, *qacE*, *qacEdelta1*, *sul1*, *sul2*, *tet(*A*)*, *tet(*B*)*, *tet(*D*)*, *tet(*O*)*
6	Typhimurium	IncFIB	*ant(3″)*, *aph(3″)*, *aph(3′)*, *bla*_TEM_, *cmlA*, *cmlB*, *dfrA*, *floR*, *qacF*, *qacL*, *sul1*, *sul2*, *sul2*, *tet(*M*)*
7	Typhimurium Dublin	IncA/C2	*aph(3″)*, *aph(6)*, *bla*_BIL_, *bla*_CFE_, *bla*_CMY_, *bla*_TEM_, *floR*, *sul1*, *sul2*, *tet(*A*)*
8	Typhimurium	IncFIB	*ant(3″)*, *lnuF*, *qnrS*, *tet(*M*)*, *tet(*O*)*, *tet(*S*)*
9	Dublin	IncA/C2	*aph(3″)*, *aph(6)*, *bla*_BIL_, *bla*_CFE_, *bla*_CMY_, *bla*_LAT_, *bla*_TEM_, *floR*, *sul1*, *sul2*, *tet(*A*)*
10	Dublin	IncA/C2	*aph(3″)*, *aph(3′)*, *aph(6)*, *bla*_BIL_, *bla*_CFE_, *bla*_CMY_, *bla*_LAT_, *bla*_TEM_, *floR*, *sul1*, *sul2*, *tet(*A*)*
11	Meleagridis	IncHI2	*aac(3)*, *ant(3″)*, *aph(3″)*, *aph(3′)*, *qacE*, *qacEdelta1*, *rmtE*, *sul1*, *sul2*, *tet(*A*)*
12	Bredeney Anatum	IncA/C2 IncR	*aph(3″)*, *aph(3′)*, *aph(6)*, *bla*_BIL_, *bla*_CFE_, *bla*_CMY_, *bla*_LAT_, *floR*, *sul1*, *sul2*, *tet(*A*)*
13	Uganda	IncHI2	*ant(3″)*, *aph(3″)*, *aph(3′)*, *bla*_CTX-M_, *bla*_TEM_, *lnuF*, *mphA*, *qacE*, *qacEdelta1*, *qacG*, *sul1*, *sul2*, *sul2I*, *tet(*A*)*, *tet(*M*)*, *tet(*S*)*

Bandage [29] was used to co-localize antimicrobial resistance genes and plasmid sequences on contigs. Antimicrobial resistance genes and plasmid sequences were detected via ABRicate [30] with the MEGARes [31] and PlasmidFinder [32] databases, respectively.

**Table 4:** Antimicrobial resistance genes associated with *Salmonella* serotypes in Texas Panhandle cattle.

Salmonella Serotype	ARGs	Odds Ratio *
Dublin	*aph(3″)*	Inf
	*aph(6)*	Inf
	*aph(3′)*	33.4
	*bla*_CMY_	Inf
	*floR*	Inf
	*sul2*	Inf
	*tet(*A*)*	Inf
Heidelberg	*ant(3″)*	Inf
	*aph(3″)*	Inf
	*aph(6)*	Inf
	*bla*_CMY_	Inf
	*dfrA*	Inf
	*floR*	56.9
	*fosA*	Inf
	*qnrB*	49.1
	*sul1*	Inf
	*sul2*	Inf
	*tet(*A*)*	Inf
	*tet(*B*)*	Inf
	*tet(*D*)*	Inf
	*tet(*O*)*	Inf
Meleagridis	*aac(2′)*	Inf
Montevideo	*ramA*	Inf

ARGs: antimicrobial resistance genes; Inf indicates completely separable serotype–genotype associations; * Odds ratios are main effects ORs and were adjusted for population structure and false discovery rate (Benjamini–Hochberg).

## Data Availability

De-identified data sets from this study are available in the SRA archives at NCBI (https://www.ncbi.nlm.nih.gov/sra/PRJNA1135485, accessed on 16 July 2024).

## References

[1] CDC Salmonella. https://www.cdc.gov/salmonella/index.html.

[2] Ferrari R.G., Rosario D.K.A., Cunha-Neto A., Mano S.B., Figueiredo E.E.S., Conte-Junior C.A. (2019). Worldwide Epidemiology of Salmonella Serovars in Animal-Based Foods: A Meta-analysis. Appl. Environ. Microbiol..

[3] Razafindrazoto C.I., Rakotomalala J.A., Randriamifidy N.H., Ralaizanaka B.M., Maherison S., Hasina Laingonirina D.H., Rakotomaharo M., Rasolonjatovo A.S., Rakotozafindrabe A.L.R., Rabenjanahary T.H. (2021). Acute infectious pancreatitis due to Salmonella typhi: Case report and literature review. JGH Open.

[4] Elouali A., Ouerradi N., Ayad G., Babakhouya A., Rkain M. (2023). Salmonella Meningitis in a Young Infant: A Case Report. Cureus.

[5] Hull D.M., Harrell E., Harden L., Thakur S. (2022). Multidrug resistance and virulence genes carried by mobile genomic elements in Salmonella enterica isolated from live food animals, processed, and retail meat in North Carolina, 2018–2019. Int. J. Food Microbiol..

[6] Medalla F., Gu W., Friedman C.R., Judd M., Folster J., Griffin P.M., Hoekstra R.M. (2021). Increased Incidence of Antimicrobial-Resistant Nontyphoidal Salmonella Infections, United States, 2004–2016. Emerg. Infect. Dis..

[7] Scharff R.L. (2020). Food Attribution and Economic Cost Estimates for Meat- and Poultry-Related Illnesses. J. Food Prot..

[8] NACMCF (2024). Response to Questions Posed by the Food Safety and Inspection Service: Enhancing Salmonella Control in Poultry Products. J. Food Prot..

[9] Tate H., Li C., Nyirabahizi E., Tyson G.H., Zhao S., Rice-Trujillo C., Jones S.B., Ayers S., M’Ikanatha N.M., Hanna S. (2021). A National Antimicrobial Resistance Monitoring System Survey of Antimicrobial-Resistant Foodborne Bacteria Isolated from Retail Veal in the United States. J. Food Prot..

[10] Carroll L.M., Wiedmann M., den Bakker H., Siler J., Warchocki S., Kent D., Lyalina S., Davis M., Sischo W., Besser T. (2017). Whole-Genome Sequencing of Drug-Resistant Salmonella enterica Isolates from Dairy Cattle and Humans in New York and Washington States Reveals Source and Geographic Associations. Appl. Environ. Microbiol..

[11] Pornsukarom S., van Vliet A.H.M., Thakur S. (2018). Whole genome sequencing analysis of multiple Salmonella serovars provides insights into phylogenetic relatedness, antimicrobial resistance, and virulence markers across humans, food animals and agriculture environmental sources. BMC Genom..

[12] Carroll L.M., Huisman J.S., Wiedmann M. (2020). Twentieth-century emergence of antimicrobial resistant human- and bovine-associated Salmonella enterica serotype Typhimurium lineages in New York State. Sci. Rep..

[13] Nichols M., Gollarza L., Sockett D., Aulik N., Patton E., Francois Watkins L.K., Gambino-Shirley K.J., Folster J.P., Chen J.C., Tagg K.A. (2022). Outbreak of Multidrug-Resistant Salmonella Heidelberg Infections Linked to Dairy Calf Exposure, United States, 2015–2018. Foodborne Pathog. Dis..

[14] Carroll L.M., Buehler A.J., Gaballa A., Siler J.D., Cummings K.J., Cheng R.A., Wiedmann M. (2021). Monitoring the Microevolution of Salmonella enterica in Healthy Dairy Cattle Populations at the Individual Farm Level Using Whole-Genome Sequencing. Front. Microbiol..

[15] Lewis G.L., Fenton R.J., Moriyama E.N., Loy J.D., Moxley R.A. (2023). Association of ISVsa3 with Multidrug Resistance in Salmonella enterica Isolates from Cattle (Bos taurus). Microorganisms.

[16] Nickodem C., Arnold A., Gehring K.B., Gill J.J., Richeson J.T., Samuelson K.L., Scott H., Smith J., Taylor T., Vinasco J. (2023). A longitudinal study on the dynamics of Salmonella enterica prevalence and serovar composition in beef cattle feces and lymph nodes and potential contributing sources from the feedlot environment. Appl. Environ. Microbiol..

[17] Velasquez-Munoz A., Castro-Vargas R., Cullens-Nobis F.M., Mani R., Abuelo A. (2023). Review: Salmonella Dublin in dairy cattle. Front. Vet. Sci..

[18] Cornell University CVM Salmonellosis: Background, Management and Control. https://www.vet.cornell.edu/animal-health-diagnostic-center/programs/nyschap/modules-documents/salmonellosis-background-management-and-control#:~:text=Overall%2C%2028%25%20of%20dairy%20farms,a%20short%20period%20of%20time.

[19] Eyler A.B., M’Ikanatha N.M., Xiaoli L., Dudley E.G. (2020). Whole-genome sequencing reveals resistome of highly drug-resistant retail meat and human Salmonella Dublin. Zoonoses Public Health.

[20] Srednik M.E., Morningstar-Shaw B.R., Hicks J.A., Mackie T.A., Schlater L.K. (2022). Antimicrobial resistance and genomic characterization of Salmonella enterica serovar Senftenberg isolates in production animals from the United States. Front. Microbiol..

[21] Marshall K.E.H., Tewell M., Tecle S., Leeper M., Sinatra J., Kissler B., Fung A., Brown K., Wagner D., Trees E. (2018). Protracted Outbreak of Salmonella Newport Infections Linked to Ground Beef: Possible Role of Dairy Cows—21 States, 2016–2017. MMWR Morb. Mortal. Wkly. Rep..

[22] Levent G., Schlochtermeier A., Ives S.E., Norman K.N., Lawhon S.D., Loneragan G.H., Anderson R.C., Vinasco J., Scott H.M. (2019). Population Dynamics of Salmonella enterica within Beef Cattle Cohorts Followed from Single-Dose Metaphylactic Antibiotic Treatment until Slaughter. Appl. Environ. Microbiol..

[23] Manishimwe R., Moncada P.M., Bugarel M., Scott H.M., Loneragan G.H. (2021). Antibiotic resistance among Escherichia coli and Salmonella isolated from dairy cattle feces in Texas. PLoS ONE.

[24] Levent G., Schlochtermeier A., Ives S.E., Norman K.N., Lawhon S.D., Loneragan G.H., Anderson R.C., Vinasco J., den Bakker H.C., Scott H.M. (2021). High-Resolution Genomic Comparisons within Salmonella enterica Serotypes Derived from Beef Feedlot Cattle: Parsing the Roles of Cattle Source, Pen, Animal, Sample Type, and Production Period. Appl. Environ. Microbiol..

[25] Taylor E.A., Ossa-Trujillo C., Vinasco J., Jordan E.R., García Buitrago J.A., Hagevoort R., Norman K.N., Lawhon S.D., Piñeiro J.M., Levent G. (2021). Use of critically important antimicrobial classes early in life may adversely impact bacterial resistance profiles during adult years: Potential co-selection for plasmid-borne fluoroquinolone and macrolide resistance via extended-spectrum beta-lactam use in dairy cattle. Lett. Appl. Microbiol..

[26] USDA Annual State Agricultural Exports Interactive Chart. https://www.ers.usda.gov/data-products/state-agricultural-trade-data/annual-state-agricultural-exports/.

[27] CLSI (2023). Performance Standards for Antimicrobial Disk and Dilution Susceptibility Tests for Bacteria Isolated from Animals.

[28] Yoshida C.E., Kruczkiewicz P., Laing C.R., Lingohr E.J., Gannon V.P., Nash J.H., Taboada E.N. (2016). The Salmonella In Silico Typing Resource (SISTR): An Open Web-Accessible Tool for Rapidly Typing and Subtyping Draft Salmonella Genome Assemblies. PLoS ONE.

[29] Wick R.R., Schultz M.B., Zobel J., Holt K.E. (2015). Bandage: Interactive visualization of de novo genome assemblies. Bioinformatics.

[30] Seemann T. Abricate. https://github.com/tseemann/abricate.

[31] Doster E., Lakin S.M., Dean C.J., Wolfe C., Young J.G., Boucher C., Belk K.E., Noyes N.R., Morley P.S. (2020). MEGARes 2.0: A database for classification of antimicrobial drug, biocide and metal resistance determinants in metagenomic sequence data. Nucleic Acids Res..

[32] Carattoli A., Zankari E., García-Fernández A., Voldby Larsen M., Lund O., Villa L., Møller Aarestrup F., Hasman H. (2014). In silico detection and typing of plasmids using PlasmidFinder and plasmid multilocus sequence typing. Antimicrob. Agents Chemother..

[33] Zhou Z., Alikhan N.F., Mohamed K., Fan Y., Achtman M. (2020). The EnteroBase user’s guide, with case studies on Salmonella transmissions, Yersinia pestis phylogeny, and Escherichia core genomic diversity. Genome Res..

[34] Veeraraghavan B., Jacob J.J., Prakash J.A.J., Pragasam A.K., Neeravi A., Narasimman V., Anandan S. (2019). Extensive drug resistant Salmonella enterica serovar Senftenberg carrying bla(NDM) encoding plasmid p5558 (IncA/C) from India. Pathog. Glob. Health.

[35] Power R.A., Parkhill J., de Oliveira T. (2017). Microbial genome-wide association studies: Lessons from human GWAS. Nat. Rev. Genet..

[36] Sul J.H., Martin L.S., Eskin E. (2018). Population structure in genetic studies: Confounding factors and mixed models. PLoS Genet..

[37] Paudyal N., Pan H., Elbediwi M., Zhou X., Peng X., Li X., Fang W., Yue M. (2019). Characterization of Salmonella Dublin isolated from bovine and human hosts. BMC Microbiol..

[38] Morris C., Wickramasingha D., Abdelfattah E.M., Pereira R.V., Okello E., Maier G. (2023). Prevalence of antimicrobial resistance in fecal Escherichia coli and Enterococcus spp. isolates from beef cow-calf operations in northern California and associations with farm practices. Front. Microbiol..

[39] Berge A.C., Hancock D.D., Sischo W.M., Besser T.E. (2010). Geographic, farm, and animal factors associated with multiple antimicrobial resistance in fecal Escherichia coli isolates from cattle in the western United States. J. Am. Vet. Med. Assoc..

[40] Yamamoto S., Iwabuchi E., Hasegawa M., Esaki H., Muramatsu M., Hirayama N., Hirai K. (2013). Prevalence and molecular epidemiological characterization of antimicrobial-resistant Escherichia coli isolates from Japanese black beef cattle. J. Food Prot..

[41] Markland S., Weppelmann T.A., Ma Z., Lee S., Mir R.A., Teng L., Ginn A., Lee C., Ukhanova M., Galindo S. (2019). High Prevalence of Cefotaxime Resistant Bacteria in Grazing Beef Cattle: A Cross Sectional Study. Front. Microbiol..

[42] University of Georgia Extension Mineral Supplements for Beef Cattle. https://extension.uga.edu/publications/detail.html?number=B895&title=mineral-supplements-for-beef-cattle.

[43] Folster J.P., Pecic G., Bolcen S., Theobald L., Hise K., Carattoli A., Zhao S., McDermott P.F., Whichard J.M. (2010). Characterization of extended-spectrum cephalosporin-resistant Salmonella enterica serovar Heidelberg isolated from humans in the United States. Foodborne Pathog. Dis..

[44] Mangat C.S., Bekal S., Avery B.P., Côté G., Daignault D., Doualla-Bell F., Finley R., Lefebvre B., Bharat A., Parmley E.J. (2019). Genomic Investigation of the Emergence of Invasive Multidrug-Resistant Salmonella enterica Serovar Dublin in Humans and Animals in Canada. Antimicrob. Agents Chemother..

[45] Srednik M.E., Lantz K., Hicks J.A., Morningstar-Shaw B.R., Mackie T.A., Schlater L.K. (2021). Antimicrobial resistance and genomic characterization of Salmonella Dublin isolates in cattle from the United States. PLoS ONE.

[46] Fricke W.F., Welch T.J., McDermott P.F., Mammel M.K., LeClerc J.E., White D.G., Cebula T.A., Ravel J. (2009). Comparative genomics of the IncA/C multidrug resistance plasmid family. J. Bacteriol..

[47] Lindsey R.L., Fedorka-Cray P.J., Frye J.G., Meinersmann R.J. (2009). Inc A/C plasmids are prevalent in multidrug-resistant Salmonella enterica isolates. Appl. Environ. Microbiol..

[48] Welch T.J., Fricke W.F., McDermott P.F., White D.G., Rosso M.L., Rasko D.A., Mammel M.K., Eppinger M., Rosovitz M.J., Wagner D. (2007). Multiple antimicrobial resistance in plague: An emerging public health risk. PLoS ONE.

[49] Akinyemi K.O., Fakorede C.O., Linde J., Methner U., Wareth G., Tomaso H., Neubauer H. (2023). Whole genome sequencing of Salmonella enterica serovars isolated from humans, animals, and the environment in Lagos, Nigeria. BMC Microbiol..

[50] Raufu I.A., Ahmed O.A., Aremu A., Ameh J.A., Timme R.E., Hendriksen R.S., Ambali A.G. (2021). Occurrence, antimicrobial resistance and whole genome sequence analysis of Salmonella serovars from pig farms in Ilorin, North-central Nigeria. Int. J. Food Microbiol..

[51] Aworh M.K., Nilsson P., Egyir B., Owusu F.A., Hendriksen R.S. (2024). Rare serovars of non-typhoidal Salmonella enterica isolated from humans, beef cattle and abattoir environments in Nigeria. PLoS ONE.

[52] Cummings K.J., Rodriguez-Rivera L.D., Norman K.N., Ohta N., Scott H.M. (2017). Identification of a Plasmid-Mediated Quinolone Resistance Gene in Salmonella Isolates from Texas Dairy Farm Environmental Samples. Zoonoses Public Health.

[53] Slowey R., Kim S.W., Prendergast D., Madigan G., Van Kessel J.A.S., Haley B.J. (2022). Genomic diversity and resistome profiles of Salmonella enterica subsp. enterica serovar Kentucky isolated from food and animal sources in Ireland. Zoonoses Public Health.

[54] Cloeckaert A., Chaslus-Dancla E. (2001). Mechanisms of quinolone resistance in Salmonella. Vet. Res..

[55] Hooper D.C., Jacoby G.A. (2015). Mechanisms of drug resistance: Quinolone resistance. Ann. N. Y. Acad. Sci..

[56] McDermott P.F., Tyson G.H., Kabera C., Chen Y., Li C., Folster J.P., Ayers S.L., Lam C., Tate H.P., Zhao S. (2016). Whole-Genome Sequencing for Detecting Antimicrobial Resistance in Nontyphoidal Salmonella. Antimicrob. Agents Chemother..

[57] Day M.R., Doumith M., Do Nascimento V., Nair S., Ashton P.M., Jenkins C., Dallman T.J., Stevens F.J., Freedman J., Hopkins K.L. (2018). Comparison of phenotypic and WGS-derived antimicrobial resistance profiles of Salmonella enterica serovars Typhi and Paratyphi. J. Antimicrob. Chemother..

[58] Katz T.S., Harhay D.M., Schmidt J.W., Wheeler T.L. (2024). Identifying a list of Salmonella serotypes of concern to target for reducing risk of salmonellosis. Front. Microbiol..

[59] Fritz H.M., Pereira R.V., Toohey-Kurth K., Marshall E., Tucker J., Clothier K.A. (2022). Salmonella enterica Serovar Dublin from Cattle in California from 1993-2019: Antimicrobial Resistance Trends of Clinical Relevance. Antibiotics.

[60] Campioni F., Vilela F.P., Cao G., Kastanis G., Dos Prazeres Rodrigues D., Costa R.G., Tiba-Casas M.R., Yin L., Allard M., Falcão J.P. (2022). Whole genome sequencing analyses revealed that Salmonella enterica serovar Dublin strains from Brazil belonged to two predominant clades. Sci. Rep..

[61] Afema J.A., Mather A.E., Sischo W.M. (2015). Antimicrobial Resistance Profiles and Diversity in Salmonella from Humans and Cattle, 2004–2011. Zoonoses Public Health.

[62] Rodriguez-Rivera L.D., Moreno Switt A.I., Degoricija L., Fang R., Cummings C.A., Furtado M.R., Wiedmann M., den Bakker H.C. (2014). Genomic characterization of Salmonella Cerro ST367, an emerging Salmonella subtype in cattle in the United States. BMC Genom..

[63] Cummings K.J., Warnick L.D., Elton M., Rodriguez-Rivera L.D., Siler J.D., Wright E.M., Gröhn Y.T., Wiedmann M. (2010). Salmonella enterica serotype Cerro among dairy cattle in New York: An emerging pathogen?. Foodborne Pathog. Dis..

[64] Fluit A.C. (2005). Towards more virulent and antibiotic-resistant Salmonella?. FEMS Immunol. Med. Microbiol..

[65] Mølbak K. (2005). Human health consequences of antimicrobial drug-resistant Salmonella and other foodborne pathogens. Clin. Infect. Dis..

[66] Burciaga S., Trachsel J.M., Sockett D., Aulik N., Monson M.S., Anderson C.L., Bearson S.M.D. (2023). Genomic and phenotypic comparison of two variants of multidrug-resistant Salmonella enterica serovar Heidelberg isolated during the 2015–2017 multi-state outbreak in cattle. Front. Microbiol..

[67] McMillan E.A., Weinroth M.D., Frye J.G. (2022). Increased Prevalence of Salmonella Infantis Isolated from Raw Chicken and Turkey Products in the United States Is Due to a Single Clonal Lineage Carrying the pESI Plasmid. Microorganisms.

[68] Fonseca M., Heider L.C., Stryhn H., McClure J.T., Léger D., Rizzo D., Dufour S., Roy J.P., Kelton D.F., Renaud D.L. (2024). Frequency of isolation and phenotypic antimicrobial resistance of fecal Salmonella enterica recovered from dairy cattle in Canada. J. Dairy Sci..

[69] Aleri J.W., Sahibzada S., Harb A., Fisher A.D., Waichigo F.K., Lee T., Robertson I.D., Abraham S. (2022). Molecular epidemiology and antimicrobial resistance profiles of Salmonella isolates from dairy heifer calves and adult lactating cows in a Mediterranean pasture-based system of Australia. J. Dairy Sci..

[70] Noyes N.R., Yang X., Linke L.M., Magnuson R.J., Cook S.R., Zaheer R., Yang H., Woerner D.R., Geornaras I., McArt J.A. (2016). Characterization of the resistome in manure, soil and wastewater from dairy and beef production systems. Sci. Rep..

[71] Carey A.M., Capik S.F., Giebel S., Nickodem C., Piñeiro J.M., Scott H.M., Vinasco J., Norman K.N. (2022). Prevalence and Profiles of Antibiotic Resistance Genes mph(A) and qnrB in Extended-Spectrum Beta-Lactamase (ESBL)-Producing Escherichia coli Isolated from Dairy Calf Feces. Microorganisms.

[72] Cao H., Pradhan A.K., Karns J.S., Hovingh E., Wolfgang D.R., Vinyard B.T., Kim S.W., Salaheen S., Haley B.J., Van Kessel J.A.S. (2019). Age-Associated Distribution of Antimicrobial-Resistant Salmonella enterica and Escherichia coli Isolated from Dairy Herds in Pennsylvania, 2013–2015. Foodborne Pathog. Dis..

[73] Pereira R.V., Siler J.D., Ng J.C., Davis M.A., Grohn Y.T., Warnick L.D. (2014). Effect of on-farm use of antimicrobial drugs on resistance in fecal Escherichia coli of preweaned dairy calves. J. Dairy Sci..

[74] Hille K., Ruddat I., Schmid A., Hering J., Hartmann M., von Münchhausen C., Schneider B., Messelhäusser U., Friese A., Mansfeld R. (2017). Cefotaxime-resistant E. coli in dairy and beef cattle farms-Joint analyses of two cross-sectional investigations in Germany. Prev. Vet. Med..

[75] Davis M.A., Hancock D.D., Besser T.E., Daniels J.B., Baker K.N., Call D.R. (2007). Antimicrobial resistance in Salmonella enterica serovar Dublin isolates from beef and dairy sources. Vet. Microbiol..

[76] Lipkens Z., Piepers S., De Vliegher S. (2023). Impact of Selective Dry Cow Therapy on Antimicrobial Consumption, Udder Health, Milk Yield, and Culling Hazard in Commercial Dairy Herds. Antibiotics.

[77] Stevens M., Piepers S., Supré K., Dewulf J., De Vliegher S. (2016). Quantification of antimicrobial consumption in adult cattle on dairy herds in Flanders, Belgium, and associations with udder health, milk quality, and production performance. J. Dairy. Sci..

[78] Tello M., Ocejo M., Oporto B., Hurtado A. (2020). Prevalence of Cefotaxime-Resistant Escherichia coli Isolates from Healthy Cattle and Sheep in Northern Spain: Phenotypic and Genome-Based Characterization of Antimicrobial Susceptibility. Appl. Environ. Microbiol..

[79] USDA Can Antibiotics Be Used in Cattle Raising?. https://ask.usda.gov/s/article/Can-antibiotics-be-used-in-cattle-raising#:~:text=Antibiotics%20may%20be%20given%20to,can%20exit%20the%20animal’s%20system.

[80] Rovira P., McAllister T., Lakin S.M., Cook S.R., Doster E., Noyes N.R., Weinroth M.D., Yang X., Parker J.K., Boucher C. (2019). Characterization of the Microbial Resistome in Conventional and “Raised Without Antibiotics” Beef and Dairy Production Systems. Front. Microbiol..

[81] Wang W., Wei X., Wu L., Shang X., Cheng F., Li B., Zhou X., Zhang J. (2021). The occurrence of antibiotic resistance genes in the microbiota of yak, beef and dairy cattle characterized by a metagenomic approach. J. Antibiot..

[82] Warburton D.W., Bowen B., Konkle A., Crawford C., Durzi S., Foster R., Fox C., Gour L., Krohn G., LaCasse P. (1994). A comparison of six different plating media used in the isolation of Salmonella. Int. J. Food Microbiol..

[83] British Standards Institution (1990). Methods for Microbiological Examination of Food and Animal Feeding Stuffs: Part 4. Detection of Salmonella.

[84] Arroyo G., Arroyo J.A. (1995). Efficiency of different enrichment and isolation procedures for the detection of Salmonella serotypes in edible offal. J. Appl. Bacteriol..

[85] Cameron A., McAllister T.A. (2016). Antimicrobial usage and resistance in beef production. J. Anim. Sci. Biotechnol..

[86] Schwarz S., Silley P., Simjee S., Woodford N., van Duijkeren E., Johnson A.P., Gaastra W. (2010). Editorial: Assessing the antimicrobial susceptibility of bacteria obtained from animals. J. Antimicrob. Chemother..

[87] D’Amato R.F., Hochstein L., Vernaleo J.R., Cleri D.J., Wallman A.A., Gradus M.S., Thornsberry C. (1985). Evaluation of the BIOGRAM antimicrobial susceptibility test system. J. Clin. Microbiol..

[88] Fader R.C., Weaver E., Fossett R., Toyras M., Vanderlaan J., Gibbs D., Wang A., Thierjung N. (2013). Multilaboratory study of the Biomic automated well-reading instrument versus MicroScan WalkAway for reading MicroScan antimicrobial susceptibility and identification panels. J. Clin. Microbiol..

[89] Fukasawa Y., Ermini L., Wang H., Carty K., Cheung M.S. (2020). LongQC: A Quality Control Tool for Third Generation Sequencing Long Read Data. G3.

[90] Kolmogorov M., Yuan J., Lin Y., Pevzner P.A. (2019). Assembly of long, error-prone reads using repeat graphs. Nat. Biotechnol..

[91] Koren S., Walenz B.P., Berlin K., Miller J.R., Bergman N.H., Phillippy A.M. (2017). Canu: Scalable and accurate long-read assembly via adaptive k-mer weighting and repeat separation. Genome Res..

[92] Nanoporetech Medaka. https://github.com/nanoporetech/medaka.

[93] Huang Y.T., Liu P.Y., Shih P.W. (2021). Homopolish: A method for the removal of systematic errors in nanopore sequencing by homologous polishing. Genome Biol..

[94] Gurevich A., Saveliev V., Vyahhi N., Tesler G. (2013). QUAST: Quality assessment tool for genome assemblies. Bioinformatics.

[95] Seppey M., Manni M., Zdobnov E.M. (2019). BUSCO: Assessing Genome Assembly and Annotation Completeness. Methods Mol. Biol..

[96] Seemann T. Snippy. https://github.com/tseemann/snippy.

[97] Tamura K., Stecher G., Kumar S. (2021). MEGA11: Molecular Evolutionary Genetics Analysis Version 11. Mol. Biol. Evol..

[98] Brynildsrud O., Bohlin J., Scheffer L., Eldholm V. (2016). Rapid scoring of genes in microbial pan-genome-wide association studies with Scoary. Genome Biol..

[99] Lees J.A., Galardini M., Bentley S.D., Weiser J.N., Corander J. (2018). pyseer: A comprehensive tool for microbial pangenome-wide association studies. Bioinformatics.

